# Incorporating evidence-based principles in medical training. Sharing experience with McMaster

**DOI:** 10.15694/mep.2018.0000269.1

**Published:** 2018-11-29

**Authors:** Silke Anna Theresa Weber, Aristides Palhares Neto, Luciana Patrícia Abbade, Jacqueline Costa Teixeira Caramori, Gilmar Reis, Rosemary Oliveira, Lehana Thabane

**Affiliations:** 1 Botucatu Medical School-UNESP; 2 Catholic University of Minas Gerais; 3 McMaster University

**Keywords:** Medical education, evidence-based teaching, innovation, medicine, research teaching

## Abstract

This article was migrated. The article was marked as recommended.

**Background:** This workshop was the second activity of the collaboration between the McMaster University, Botucatu Medical School- São Paulo State University (UNESP) and Pontifical Catholic University of Minas Gerais – PUC Minas that took place in Botucatu, Brazil between March 27th to 28th 2017.

**Aims:** Its prime purpose was to share with the Brazilian professors and students how to include evidence-based concepts in their daily teaching activities.

**Methods:** The participants were involved and guided in discussions on how to explore evidence-based techniques to improve their understanding and their willingness to include new teaching strategies in the future.

**Results:** A final evaluation survey completed by the participants indicated that they were highly satisfied with the workshop experience and that they gained an enhancement of knowledge about evidence-based medicine.

**Conclusion:** Participants had an increase in their self-confidence to implementevidence-based concepts in their future lecture programs.

## Introduction

Evidence-based practice is defined as the conscientious, explicit and judicious use of current best evidence in decision-making about individual patient care. It is a process of systematically asking, acquiring, appraising, and applying research findings as the basis for clinical decisions ( Guyatt and Busse, 2002).

In order to promote better understanding of evidence-based strategies in teaching and research, a group of professors of Botucatu Medical School-São Paulo State University (Unesp) and Pontifical Catholic University of Minas Gerais - PUC Minas initiated a collaborative process with McMaster University-Canada. After the first meeting held in Canada in 2015, the group decided to share their knowledge and discussions with professors, lecturers and students from their home universities in an informative workshop. The workshop unveiled principles of evidence-based learning in teaching and research, emphasizing the necessary importance of continuous actions to promote planning of strategies for new teaching models and for research activity during medical training.

The purpose of this report is to provide an account of the lectures and discussions held during the workshop, and to report on the outcome results of an assessment completed by the participants at the end of the workshop.

## Workshop Report

### Location

The workshop took place from March 27 ^th^ to 28 ^th^, 2017 at the Botucatu Medical School-UNESP, Botucatu, São Paulo - Brazil.

### Aims

The purpose of the workshop was to reveal different aspects of evidence-based methodology in active teaching methods in medical education, intending to enhance the knowledge of a group of Brazilian medical faculty professors, in order to facilitate the introduction of evidence-based principles in their home curriculum. The workshop also aimed to strengthen the cooperation between the participating universities in medical education and in global health capacity building.

### Participants

The organizing committee invited lecturers, physicians and researchers from both medical schools who are linked to the collaboration program, and also invited and welcomed graduate and undergraduate students from the two universities.

### Facilitators

The workshop was facilitated by Lehana Thabane, Professor, Associate Chair of the Department of Biostatistics and Epidemiology CE&B, McMaster University, who possesses vast experience in evidence-based medicine in medical training. The workshop was also facilitated by Brazilian professors, Aristides Palhares Neto, Jacqueline Caramori, Luciana Abbade, Silke Weber, and Gilmar Reis. This group of professors had previously participated in the first workshop held at McMaster University in Hamilton, ON, Canada in 2015.

### Program

During the two-day program, each period was reserved for a one-hour lecture followed by group discussions that were determined by previously elaborated questions and activities from the facilitators. To promote active participation, the participants were divided into smaller groups of up to 10 people. The facilitating professors created each group by including a mix of participating professors, physicians and students. Four topics were selected based on their importance for medical training and medical faculties:

#### 1. The importance of evidence-based medicine principles

The primary objective of this session was to discuss the importance of teaching evidence-based medicine (EBM) principles that health practitioners need to learn to assess the quality of evidence to guide their clinical or health decisions ( Straus and Sackett, 1998; Green *et al*., 2000). This lecture provided an overview of the principles of EBM, using three principles to illustrate the issues: 1) EBM should be based on evidence from large, well designed trials; 2) EBM should be skeptical of evidence from subgroup analyses in trials; and 3) EBM should be based on carefully scrutinized evidence from both RCTs and observational studies. Some examples of the discrepancies between the results of meta-analysis based on small RCTs versus the results of a single large trial were provided to illustrate the first principle ( Alderson and Roberts, 1997; Roberts *et al*., 2004). The lecture also discussed the motivations for doing subgroup analyses in trials and why such analyses are generally unreliable ( Rothwell, 2005). Some guidance was provided about resources that one could use to assess the credibility of subgroup findings from trials ( Brookes *et al*., 2004). The session also discussed several cases where the results of observational studies showed similar results to those of RCTs on the same topic ( Singh-Grewal, Macdessi and Craig, 2005) and examples where the differences were also shown ( Egger, Schneider and Davey Smith, 1998). Reasons for similarity or differences in findings between the two types of evidence were discussed. Overall, the message was that while observational studies have some important role in EBM, they need to be approached with caution because there are many situations where reviews of OS can mislead because confounding, selection bias and measurement error often distort the findings. They present the danger in producing very precise, but spurious results. However, there are also several instances where reviews of well-designed OS and those of RCTS often show similar results in both the direction and magnitude of effects.

The group discussion was based on the reading of previously chosen articles with different study designs, such as a large RCT, a meta-analysis of small RTCs and observational study. The participants were asked to indicate which article was plausible, which factors contributed to their positive analysis, which ones had negative interpretation, and which ones would make them change their practice based on the results, and why.

Key learnings from the session

• To practise EBM, one needs to understand
  • How evidence is generated
  • Different forms of evidence
  • How to evaluate the quality of the evidence that inform practice guidelines
• Training in EBP principles in medical training is an imperative

#### 2. Insertion of clinical reasoning based on evidence

The second lecture discussed two topics: i) The role of pilot studies in medical training, and ii) Framing research questions. The coverage included practice exercises illustrating how one could incorporate the issues in the medical curriculum as part of training on research and EBM priciples.

Coverage of the role of pilot studies, included defining the importance of a small-scale investigational study to test feasibility or find possible effects and associations that may be worthy for testing in larger studies ( Thabane *et al*., 2010). The discussion also covered multiple terms such as Pilot, Feasibility, and Proof-of-concept studies, among others, which are used without clear distinction ( Eldrige *et al*., 2016). An emphasis was placed on the importance of planning good pilot studies and how to avoid common misconceptions ( Lancaster, Dodd and Williamson, 2004). Lastly, we discussed the CONSORT (Consolidated Standards of Reporting Trials) extension for pilot trials used as a tool to guide the transparent reporting of the pilot trials ( Thabane *et al*., 2016). In the second part of this lecture, the structure of the research question based on PICOT (P=patients, I= intervention, C=control, O=outcome, T=time) was presented, as well as the important aspects of FINER(F=feasible, I=interesting, N=new, E=ethical, R=relevant) ( Thabane *et al*. 2009).

The session also included some group activities. Workshop participants were divided into groups. Some groups were asked to identify the research questions of several articles, looking for the PICOT structure, and if not present, how it could be reframed. The other groups were asked to discuss the necessity of a pilot study to form a new research problem, and to identify some of the most important regional questions to be investigated based on the principles of PICOT and FINER.

Key learnings from the session: the participants were trained to have an awareness of inadequate structuring of research questions, and an awareness that problems are not previously defined, which causes a lack of focusing fully on the problem or issue. They were also trained to build and create research questions, based on good quality principles.

#### 3. Nurture a culture of healthy collaborations and research productivity in research- the evidence-based approach

This session included discussion on the importance of capacity-building in evidence-based practice (EBP) to individuals and society. Professor Thabane presented evidence to support three linked hypotheses:

*Hypothesis 1*: Capacity-building in research leads to better EBP;

*Hypothesis 2*: EBP leads to better health; and

*Hypothesis 3*: Good health is associated to higher productivity.

Using these as the basis, he shared some practical strategies that can help to cultivate a culture of healthy collaborations and inclusiveness in research. These include:

• Build and strengthen “people-centered” research leadership at all levels
• Lead by example
• Foster student-focused training
• Enhance research productivity
• Create and enrich a culture of international collaborations for the whole department
• Foster good global citizenship-collective responsibility
• Public relations
• Integrate key principles of collaboration in the training of researchers

Finally, he shared his McMaster experiences on strategies and examples of how research teams can enhance the research productivity. See Table 1 for examples.

**Table 1.** How research teams can enhance research productivity

Strategy	Examples
Be inclusive for authorship, pre-defining criteria	Inclusiveness by contributions of conception, design, acquisition, analysis and /or interpretation of data, drafting and critical review of the manuscript (ICME, 2000).
Publish i) the Protocol and the Results paper of the systematic review, and, ii) the protocol and results paper of the study.	Protocol: Morfaw *et al*., 2012. Male participation in prevention programs of mother to child transmission of HIV: a protocol for a systematic review to identify barriers, facilitators and reported interventions. Research: Morfaw *et al*., 2013. Male involvement in prevention programs of mother to child transmission of HIV: a systematic review to identify barriers and facilitators.
Write the methodological paper	Thabane *et al*., 2013. A tutorial on sensitivity analysis in clinical trials: the what, why, when and how.
Share academic process/experience	Sharing mentoring experience: Odueyungbo and Thabane, 2012. Mentoring in biostatistics: some suggestions for reform. Sharing a workshop experience: Mbuagbaw, Thabane and Ongolo-Zogo, 2013. Setting the stage for randomized controlled trials in Cameroon: a workshop report.
Set goals of research productivity	Foster mentorship, time management, stress management, effectiveness of meetings and writings. Sackett, 2001. On the determinants of academic success.

Key Learnings from the session: the participants learnt different strategies to cultivate a positive culture of health research and collaborations; and methods to enhance research productivity among their research teams.

#### 4. Teaching models in home curriculum and how to insert new models

Three professors of the home institution presented the Brazilian experiences on how to include evidence-based medicine in the local medical curriculum, which are also the current proposals for a new curriculum to be implemented. New concepts such as integration of research activities during daily routines were demonstrated as seen in Table 2.

**Table 2.** Experiences of the Brazilian university of evidence-based strategies in the local medical curriculum

Name.university	Strategy	Experience
Prof. Dr. Joelcio Abbade, FMB-UNESP	Promotion of discussions with the stake holders bringing new concepts of innovative teaching models	Development of a new curriculum, incorporation regular promotion of workshops discussing innovative teaching strategies
Prof. Dr Vânia Nunes, FMB-UNESP	Evaluation of the quality of evidence	Group discussions of scientific literature (pre-selected article) related to a patient (grand round)
Prof. Adj. Carlos Magnus Fortaleza, FMB-UNESP	Inclusion of research activities during the undergraduate course	Strategy of the medical curriculum: A period per week is directed for supervised scientific activities (extra credit account)

Conclusion: The exposition of curricular integration of evidence-based activities strengthened the feasibility of utilizing innovation in medical teaching.

#### Evaluation

**Figure 1.**
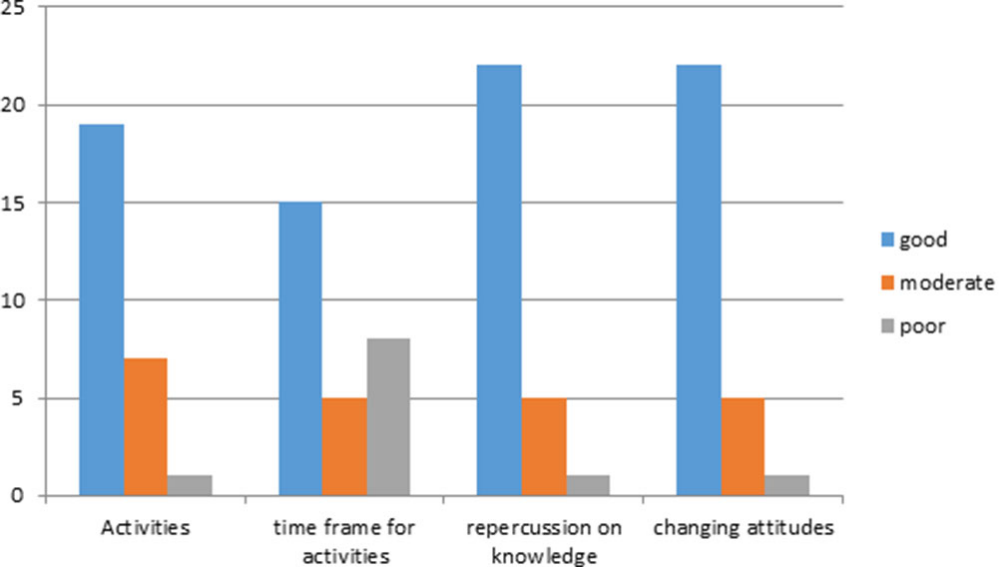
Graph of answers of the assessment considering activities, timeframe, repercussion of the own knowledge and likeliness of changing their attitudes.

At the end of the workshop, the participants completed an assessment evaluating the following ( Figure 1):

- a) activities,

- b) time frame for activities,

- c) repercussion on their own knowledge about evidence based strategies,

- d) the likeliness of changing their attitudes in teaching and research activities.

From the evaluation results, 28 out of 35 participants returned a completed questionnaire indicating that the workshop discussed important themes for their career. In essence, 17.9% reported language difficulties, 86% classified the lectures as very good and 14% as good. In total, all participants rated the workshop as highly satisfactory and indicated that they gained an improvement of knowledge about evidence-based medicine, feeling more self-confident in implementing the evidence-based concepts in their future lecture programs.

## Conclusion

The workshop successfully reached its objectives in giving the participants awareness and confidence for future implementation and application of the knowledge obtained about evidence-based principles of teaching, research and planning of the medical curriculum and health strategies. The workshop was rated by the participants as highly satisfactory on the follow-up assessment.

## Take Home Messages

• This workshop was the second activity of the collaboration between the McMaster University, Botucatu Medical School- São Paulo State University (UNESP) and Pontifical Catholic University of Minas Gerais – PUC Minas.
• Its purpose was to share how to include evidence-based concepts in daily teaching activities.
• Participants were involved and guided in discussions on how to explore evidence-based techniques.
• A final evaluation survey indicated that participants were highly satisfied with the workshop experience, enhanced their knowledge about evidence-based medicine, and increased their self-confidence to implement evidence-based concepts in the future.

## Notes On Contributors

Silke Weber, MD (UNESP – Botucatu Medical School), PhD (UNESP – Botucatu Medical School); President, International Office of UNESP – Botucatu Medical School; Associate Professor, Department of Ophthalmology, Otolaryngology and Head and Neck Surgery of UNESP – Botucatu Medical School. She focuses on actions promoting the internationalization of the Medical School, which create opportunities for innovation in medical education and integrate the insertion of the faculty in the Global Health.

ORCHID: https://orcid.org/0000-0003-3194-3039

Aristides Augusto Palhares Neto, MD (Jundiaí Medical School), PhD (UNESP – Botucatu Medical School). Assistant Professor, Department of Surgery and Orthopedics at UNESP – Botucatu Medical School. As a Plastic Surgeon he focuses in the assistance of craniofacial anomalies. At the Medical School he is enrolled in the discussion of new models of teaching and assessment in medical education.

ORCHID: https://orcid.org/0000-0002-3484-862X

Luciana Patricia Fernandes Abbade, graduated at Medicina from Universidade Estadual Paulista Júlio de Mesquita Filho (1993), master degree at Surgery from Universidade Estadual Paulista Júlio de Mesquita Filho (2001) and ph.d. at Surgery from Universidade Estadual Paulista Júlio de Mesquita Filho (2006). She received training on evidenced based techniques at McMaster University, implementing this strategies in her teaching and research models.

ORCHID: https://orcid.org/0000-0002-0334-2079

Jacqueline Costa Teixeira Caramori, MD (Universidade Federal do Pará), PhD (UNESP – Botucatu Medical School). Associate Professor, Department of Internal Medicine at UNESP – Botucatu Medical School. Chair, Peritoneal Dialysis Unit of the University Hospital of São Paulo State Secretariat of Health. Coordinator of the Medical Program at UNESP – Botucatu Medical School. As the coordination of the Medical Program, she leaded the discussion and the implementation of the new curriculum of the faculty.

ORCHID: https://orcid.org/0000-0002-0093-9515

Gilmar Reis, PHYSICIAN graduated at Universidade Federal de Minas Gerais em Belo Horizonte, MG, 1989RESEARCH FELLOW and MASTER DEGREE on cardiovascular diseases at University of Michigan, Ann Arbor MI, USA (Tutor: Prof. William Floyd Armstrong at 2003/2004. PhD in Cardiology at Heart Institute, University of São Paulo Medical School, São Paulo, SP in 2001. ASSOCIATE PROFESSOR of medicine Catholic University of Minas Gerais since 2.003.

ORCHID: https://orcid.org/0000-0002-4847-1034

Rosemary Oliveira, certified Clinical Research Associate by McMaster University, former project manager at McMaster University, currently acting as the coordinator of scientific research at CARDRESEARCH “Cardiologia Assistencial e de Pesquisa”.

Dr Lehana Thabane is a Professor of Biostatistics and Associate Chair of the Department of Health Research Methods, Evidence and Impact ( *Formerly the Department of Clinical Epidemiology and Biostatistics*), Associate member of the Departments of Pediatrics and Anesthesia at McMaster University (Hamilton, Ontario, Canada). He is the Director of Biostatistics at St Joseph’s Healthcare—Hamilton (Ontario, Canada) and Senior Scientist at the Population Health Research Institute of the Hamilton Health Sciences and McMaster University.

ORCHID: https://orcid.org/0000-0003-0355-973
