# Supplementary material for: Incorporating evidence-based principles in medical training. Sharing experience with McMaster
Source: MedEdPublish (2016). 2018 Nov 29;7:269. [Version 1] doi: 10.15694/mep.2018.0000269.1 (PMC10712007; doi:10.15694/mep.2018.0000269.1)
Supplement: Peer review history

# Reviewer report: Reviewer response for version 1

**DOI:** 10.21956/mep.19715.r29506

**Published:** 2018-12-14

**Authors:** Megan Anakin

**Affiliations:** 1 University of Otago

This review has been migrated. The reviewer awarded 4 stars out of 5 Thank you for inviting me to preview this article. Like the other two reviewers, I appreciated the clear descriptions of the evidence-based principles and how they were taken from educators in Canada applied by educators in Brazil to enhance their teaching practice. For a future article, I am wondering if the authors might like to consider emphasising the exchange of ideas that occurred during the meeting or in a follow up meeting. Perhaps you might consider signposting the exchange in the title with something like “Sharing experience between McMaster University, Canada and Sao Paulo State University, Brazil”. This framing of the article will pique the interest of readers from all parts of the world and encourage them to consider how the evidence-based principles and collaborative learning described in the article might apply to their own teaching situation.

Reviewer Expertise:

NA

No decision status is available

# Reviewer report: Reviewer response for version 1

**DOI:** 10.21956/mep.19715.r29505

**Published:** 2018-12-01

**Authors:** BALAJI ARUMUGAM

**Affiliations:** 1 TAGORE MEDICAL COLLEGE AND HOSPITAL

This review has been migrated. The reviewer awarded 4 stars out of 5 Thanks for giving the opportunity to review this article. This article presented the results of the successful workshop on evidence based principles in medical training. The objectives and the methodology of conduction of the workshop were very much in line and the results were expressed clearly in the article. The sessions were well conducted with the lecture followed by discussion on four divided topics with specific tasks. I congratulate the authors for sharing their experience of a workshop intended with a good change in the medical training using evidence based principles for the lecturers, physicians and researchers is a great move which can be followed by the other institutes taking this as a good example. The change in attitude among the majority of the workshop participants is evident with the results shown in the graph. The area which I liked the most is the first session on key leaningsTo practise EBM, one needs to understando How evidence is generatedo Different forms of evidenceo How to evaluate the quality of the evidence that inform practice guidelinesTraining in EBP principles in medical training is an imperativeGood work…

Reviewer Expertise:

NA

No decision status is available

# Reviewer report: Reviewer response for version 1

**DOI:** 10.21956/mep.19715.r29507

**Published:** 2018-11-29

**Authors:** Trevor Gibbs

**Affiliations:** 1 AMEE

This review has been migrated. The reviewer awarded 4 stars out of 5 Thank you asking me to review this paper which covers the important principle of evidence-based medicine and how it can be taught and incorporated into daily care. I enjoyed reading the paper and thought that the activity was well described, with the activities being very appropriate. In fact I thought that there was two elements to this paper- one being how to perhaps teach EBM and the other being how to use resources from another part of the world. I think that the evaluation was at a low level- perhaps appropriately at this moment in time and I would hope that the authors will consider a follow up evaluation on the participants to see how much they have used their new learning one back into their specific posts.Another thing that I think the authors missed the opportunity of doing was to explore how well EBM travels? I wonder if EBM activities from a relatively well resource area i.e. Canada, can be adopted in a less well resourced country, such as in South America.These practical approaches to medical education are well called for and I would encourage the authors to explore more in this fascinating area.All those involved in sharing their resources in teaching should read this paper.

Reviewer Expertise:

NA

No decision status is available
